# Geranylgeranyl Transferase-I Knockout Inhibits Oxidative Injury of Vascular Smooth Muscle Cells and Attenuates Diabetes-Accelerated Atherosclerosis

**DOI:** 10.1155/2020/7574245

**Published:** 2020-08-10

**Authors:** Guo-Ping Chen, Jian Yang, Guo-Feng Qian, Wei-Wei Xu, Xiao-Qin Zhang

**Affiliations:** ^1^Department of Endocrinology, The First Affiliated Hospital, College of Medicine, Zhejiang University, Hangzhou, Zhejiang 310003, China; ^2^Institute of Cardiology, The First Affiliated Hospital, College of Medicine, Zhejiang University, Hangzhou, Zhejiang 310003, China; ^3^Department of Respirology, Zhejiang Provincial People's Hospital, People's Hospital of Hangzhou Medical College, Hangzhou, Zhejiang 310014, China

## Abstract

The proliferation of vascular smooth muscle cells (VSMCs) induced by oxidative injury is one of the main features in diabetes-accelerated atherosclerosis. Geranylgeranyl transferase-I (GGTase-I) is an essential enzyme mediating posttranslational modification, especially the geranylgeranylation of small GTPase, Rac1. Our previous studies found that GGTase-I played an important role in diabetes-accelerated atherosclerosis. However, its exact role is largely unclear. In this study, mouse conditional knockout of VSMC GGTase-I (Pggt1b^*Δ*/*Δ*^ mice) was generated using the CRISPR/Cas9 system. The mouse model of diabetes-accelerated atherosclerosis was induced by streptozotocin injections and an atherogenic diet. We found that GGTase-I knockout attenuated diabetes-accelerated atherosclerosis in vivo and suppressed high-glucose-induced VSMC proliferation in vitro. Moreover, after a 16-week duration of diabetes, Pggt1b^*Δ*/*Δ*^ mice exhibited lower *α*-smooth muscle actin (*α*-SMA) and nitrotyrosine level, Rac1 activity, p47phox and NOXO1 expression, and phospho-ERK1/2 and phosphor-JNK content than wild-type mice. Meanwhile, the same changes were found in Pggt1b^*Δ*/*Δ*^ VSMCs cultured with high glucose (22.2 mM) in vitro. In conclusion, GGTase-I knockout efficiently blocked diabetes-accelerated atherosclerosis, and this protective effect must be related to the inhibition of VSMC proliferation. The potential mechanisms probably involved interfering Rac1 geranylgeranylation, inhibiting the assembly of NADPH oxidase cytosolic regulatory subunits, reducing oxidative injury, and decreasing ERK1/2 and JNK phosphorylation.

## 1. Introduction

Patients with diabetes exhibit an increased susceptibility to develop a wide range of atherosclerotic diseases by 2-4-fold, especially the stroke and myocardial infarction, which account for the majority of deaths and disability in diabetes patients [1–3]. Atherosclerosis occurs earlier and with greater severity in the population with diabetes [1–3] and is characterized pathologically by endothelial cell injury, proliferation and migration of vascular smooth muscle cells (VSMCs), and thickening of intima [4, 5]. However, the exact mechanisms responsible for this diabetes-accelerated atherosclerosis have remained elusive.

Geranylgeranyl transferase-I (GGTase-I) is one of the key enzymes mediating protein isoprenylation during the posttranslational modification, especially the geranylgeranylation of small GTPase, Rac1 [6]. Previous studies on GGTase-I mainly focused on the regulation of cell growth and differentiation via the Rac1 pathway and have been proved to be closely related to the occurrence, invasion, and metastasis of various malignant tumors [7]. Moreover, in recent years, the research on GGTase1-I in vascular remodeling has been gradually carried out and attracted attention [8, 9]. Zuckerbraun et al. found that GGTase-I inhibitor (GGTI-298) could alleviate carotid intimal thickening after balloon injury in SD rats [8]. Khan et al. reported that GGTase-I deficiency in mouse macrophages slowed down the progression of atherosclerosis [9]. But the exact role of GGTase-I in diabetes-accelerated atherosclerosis has not been involved, and further research is needed.

In recent years, our group has conducted several studies on GGTase-I and achieved some research results [10–12]. We found that in rats with abdominal aortic coarctation, the proximal aortic media (mainly VSMCs) proliferated and hypertrophied, accompanying with upregulated expression of GGTase-I [10]. It was also found that with the growth of age, VSMCs from aortic media of spontaneously hypertensive rats proliferated remarkably, the expression of GGTase-I increased, and the activity of reactive oxygen species (ROS) and NADPH oxidase (NOX) amplified gradually [11]. All these results suggest that GGTase-I plays an important role in the proliferation of VSMCs, at least in part via regulating the oxidative stress pathway. Furthermore, our latest study revealed that in diabetes-accelerated atherosclerosis, VSMCs proliferated and GGTase-I overexpressed simultaneously [12]. Then, we speculate that GGTase-I may participate in the process of diabetes-accelerated atherosclerosis by regulating the proliferation of VSMCs, but the proper mechanisms remain to be explored.

Therefore, the present study was designed to determine whether/how GGTase-I regulated oxidative stress, affecting the proliferation of VSMCs, thereby participating in the progress of diabetes-accelerated atherosclerosis.

## 2. Materials and Methods

### 2.1. Animal Models

All animal procedures conformed to the Guide for the Care and Use of Laboratory Animal published by the US National Institutes of Health (NIH Publication no. 85-23, revised 1996) and the guidelines of the Animal Care and Use Committee of Zhejiang University. Animals were maintained on a 12 h light-dark cycle and stable environmental temperature (23°C) in a pathogen-free environment with free access to chow and water. All chemicals and reagents were purchased from Sigma (St. Louis, MO, USA), unless otherwise stated.

To produce mice lacking GGTase-I in VSMCs, Pggt1b conditional knockout mice were generated using the CRISPR/Cas9 system in Shanghai Biomodel Organism Science & Technology Development Co. Briefly, Cas9 mRNA and guide RNA for Pggt1b third exon were obtained by transcription in vitro. Homologous recombinant vectors (donor vectors) were constructed by In-Fusion cloning, including 0.7 kb 5′ homology arm, 2.0 kb flox region, and 4.0 kb 3′ homology arm. Cas9 mRNA, guide RNA, and donor vectors were microinjected into the fertilized eggs of C57BL/6J mice. The resulting homologous recombinant mice were bred with C57BL/6J mice, and heterozygous offsprings were identified by genomic PCR. After backcrossed with C57BL/6J, heterozygous mice (Pggt1b^flox/+^) were mated with SM22*α*-Cre transgenic mice to generate Pggt1b conditional knockout mice (Pggt1b^flox/flox^Cre, and defined as Pggt1b^*Δ*/*Δ*^). Littermate control Pggt1b^+/+^Cre, Pggt1b^flox/+^, Pggt1b^+/+^, and Pggt1b^flox/flox^ were indistinguishable in phenotype and defined as wild type. Genetic deletion of Pggt1b was confirmed at the RNA and protein level by real-time PCR and western blotting, respectively, as previously described [12, 13].

The mouse model of diabetes-accelerated atherosclerosis was induced as previously described [12]. In brief, male eight-week Pggt1b^*Δ*/*Δ*^ and wild-type mice were rendered diabetes by i.p. daily injection of streptozotocin (STZ, S0130, Sigma-Aldrich, St. Louis, MO, USA) at a dose of 40 mg/kg for 5 days; nondiabetic animals received the vehicle (citrate buffer; 0.05 mol/l, pH: 4.5). STZ-injected mice were also fed with an atherogenic diet (21% fat and 0.15% cholesterol, wt/wt), to accelerate the progression of atherosclerosis by adding hyperlipidemia to hyperglycemia. Two weeks after the fifth STZ injection, fasting blood glucose (FBG) levels were measured in venous blood drawn from the tail by using a CONTOUR glucose meter (Bayer, Mishawaka, IN, USA). Mice with FBG over 13.9 mM were considered diabetic. Accordingly, mice were divided into four groups: diabetic Pggt1b^*Δ*/*Δ*^, nondiabetic Pggt1b^*Δ*/*Δ*^, diabetic wild type, and nondiabetic wild type. After a 16-week duration of diabetes, animals were sacrificed by cervical dislocation, their blood samples were collected, and their organs were rapidly dissected. The animal work was completed in the Central Laboratory of the First Affiliated Hospital of Zhejiang University. All assessments were performed by two investigators in a blinded manner.

### 2.2. Glucose and Lipid Measurements

FBG levels were evaluated as described above. The levels of serum total cholesterol (TC), high-density lipoprotein cholesterol (HDL-C), low-density lipoprotein cholesterol (LDL-C), and triglyceride (TG) were measured by commercial enzymatic methods (test kits from Shanghai Rongsheng Biotech, Inc., Shanghai, China).

### 2.3. Morphometric Analysis of Aortic Lesions

Aortic lesions were evaluated by en face analysis of the whole aorta and by cross-sectional analysis as previously described [14, 15]. For the en face analysis, the whole aorta was dissected out, opened longitudinally from the heart to the iliac arteries, and stained with oil red O. The total aortic surface area and the lesion area were analyzed, and the ratio of lesion area to total surface was calculated. For cross-sectional analysis, the aorta was dissected, fixed in 10% neutral formalin, embedded in paraffin, and sequentially stained with hematoxylin and eosin. Lesion areas per section were counted by taking the average of 6 sections spaced 30 *μ*m apart, beginning at the base of the aortic root. Media thickness at 10 different points of the thoracic aorta was measured and calculated. The morphometric analysis above was performed with Image-Pro Plus 6.0.

### 2.4. Immunohistochemical and Immunofluorescence Staining

Fresh-frozen aortic cryosections (10 *μ*m thick) were stored at -20°C until ready for staining. Smooth muscle cells in lesions were analyzed immunohistochemically with an antibody against *α*-smooth muscle actin (*α*-SMA, 1 : 2000, ab5694, Abcam). Lesion nitrotyrosine protein expression, a marker for oxidative stress in atheromatous lesions, was assessed by immunofluorescence staining with an antibody against nitrotyrosine (1 : 50, GTX41979, GeneTex Inc). The number of cells displaying specific staining was scored in a blinded manner.

### 2.5. Cell Culture and Treatments

VSMCs were isolated from thoracic aortic explants as previously described [13]. In brief, aortic explants from male eight-week-old Pggt1b^*Δ*/*Δ*^ and wild-type mice were cultured in Dulbecco's modified Eagle's medium (DMEM; Gibco, Grand Island, NY, USA) supplemented with 10% fetal bovine serum (FBS; Gibco) and maintained at 37°C in a humidified atmosphere of 5% CO_2_ and 95% air. After 2 weeks, cells migrating onto the tissue culture dish were collected by trypsinization and subcultured successively. The identity of the VSMCs was determined by the positive immunocytochemistry reactivity to smooth muscle-specific *α*-actin. To ensure the consistency of results, passages 5-12 of VSMCs were used for experiment. According to our previous report [12, 13], VSMC proliferation was induced by high glucose (22.2 mM) for 72 h, while the control cells were treated with normal glucose (5.6 mM). Mannitol was used as an osmotic control. VSMCs from wild-type mice were also cultured in the presence of selective GGTase-I inhibitor (GGTI-286, 10 *μ*M) or Rac1 inhibitor (100 *μ*M) for 24 h.

### 2.6. Cell Proliferation Assay

After above treatments, VSMC proliferation was measured by 3-[4,5-dimethylthiazol-2-yl]-2,5-diphenyltetrazolium bromide (MTT) assay as described previously [12, 13]. Cells were cultured in 96-well plates (5 × 10^3^ cells/well). After synchronization for 24 h, different treatments as stated above were given. Then, the cells of 96 wells were incubated with 100 *μ*l of 0.5 mg/ml MTT at 37°C for 4 h, washed with cold PBS, and lysed with 100 *μ*l of DMSO. After the insoluble crystals were completely dissolved, the optical density of each well was immediately measured at 570 nm using an automatic microplate reader (Molecular Devices, Sunnyvale, CA, USA).

### 2.7. Measurement of ROS In Vitro

Hydrogen peroxide (H_2_O_2_) was measured in VSMCs using the Amplex Red Hydrogen Peroxide/Peroxidase Assay Kit following the manufacturer's instructions (A22188, Invitrogen Molecular Probes, Eugene, OR, USA). In short, cells seeded in 6-well plates received different treatments as stated above prior to protein harvest. 20 *μ*l of whole-cell preparations, standards, and blank were assayed in triplicate in black 96-well plates after the addition of prewarmed (37°C) working solution containing 100 *μ*M Amplex Red reagents and 0.2 U/ml horseradish peroxidase. Fluorescence intensity was measured in 30 min intervals at 37°C on the fluorescence microplate reader at 544 nm excitation/590 nm emission. Data from the 120 min time point are presented as nmol of H_2_O_2_ standardized to protein concentration.

Moreover, H_2_O_2_ in VSMCs was also detected by 6-carboxy-2′,7′-dichlorodihydrofluorescein diacetate (carboxy-H_2_DCFDA, C2938, Invitrogen Molecular Probes, Eugene, OR, USA) fluorescence. Briefly, VSMCs were given different treatments as stated above. Then, cells were washed with PBS, trypsinized, and resuspended. 200 *μ*l of the resuspension was added into a black 96-well microplate and placed into an incubator at 37°C for 24 h. On the day of the experiment, cells were washed with PBS; 100 *μ*l prewarmed (37°C) loading buffer containing 10 *μ*M carboxy-H_2_DCFDA was added to each well and loaded for 40 min at 37°C. Each well was washed with PBS twice, and 100 *μ*l of PBS was added and immediately read with the fluorescence microplate reader (495 nm/525 nm) every 5 min for 60 min.

### 2.8. Determination of Activation Status of Rac1

As described previously [13], Rac1 activation was determined from tissue or cell lysates using the Rac1 G-LISA Activation Assay Kit (BK128, Cytoskeleton, Denver, CO, USA) according to the manufacturer's instruction. The signal was measured at 490 nm with an automatic microplate reader. Results are expressed as fold increase in activity compared with the control group.

### 2.9. Western Blot Analysis

The membrane protein extractions from the aortic media (VSMCs were the only cell type in this layer) or cultured VSMCs were carried out by the Plasma Membrane Protein Isolation Kit (Abcam, UK) according to the manufacturer's instructions. Total proteins were also extracted, and western blot was performed as described in our previous reports [12, 13]. The expressions of NOX subunits and mitogen-activated protein kinase (MAPK) signaling pathway were detected using their specific antibodies: anti-NOX1 (1 : 2000, ab55831, Abcam, Cambridge, UK), anti-NOX2 (1 : 500, ab80508, Abcam, Cambridge, UK), anti-p47phox (1 : 1000, ab795, Abcam, Cambridge, UK), anti-NADPH oxidase organizer 1 (anti-NOXO1, 1 : 500, sc-390927, Santa Cruz Biotechnology Co., Ltd., Japan), anti-Rac1 (1 : 1000, sc-95, Santa Cruz Biotechnology Co.), anti-p38 (1 : 1000, ab31828, Abcam), anti-phospho-p38 (p-p38, 1 : 1000, ab47363, Abcam), anti-ERK1/2 (1 : 1000, ab17942, Abcam), anti-phospho-ERK1/2 (p-ERK1/2, 1 : 1000, 9101, Cell Signaling Technology, Inc., Danvers, MA, USA), anti-JNK (1 : 1000, ab179461, Abcam), and anti-phospho-JNK (p-JNK, 1 : 1000, ab124956, Abcam). To ensure equal protein loading, *β*-actin (1 : 5000, ab8226; Abcam) and Na^+^/K^+^-ATPase (1 : 100000, ab76020, Abcam) were used as loading controls for the cytoplasm and plasma membrane, respectively.

### 2.10. Statistical Analysis

Results were expressed as mean ± standard errors of mean (SEM). All analyses were performed with SPSS (version 13.0; SPSS, Inc., Chicago, IL, USA). A two-way ANOVA followed by the Bonferroni post hoc test was used to determine significant differences between groups. Differences were considered statistically significant at a value of *P* < 0.05.

## 3. Results

### 3.1. Generation and Validation of Conditional GGTase-I Knockout Mice

**Figure 1:**
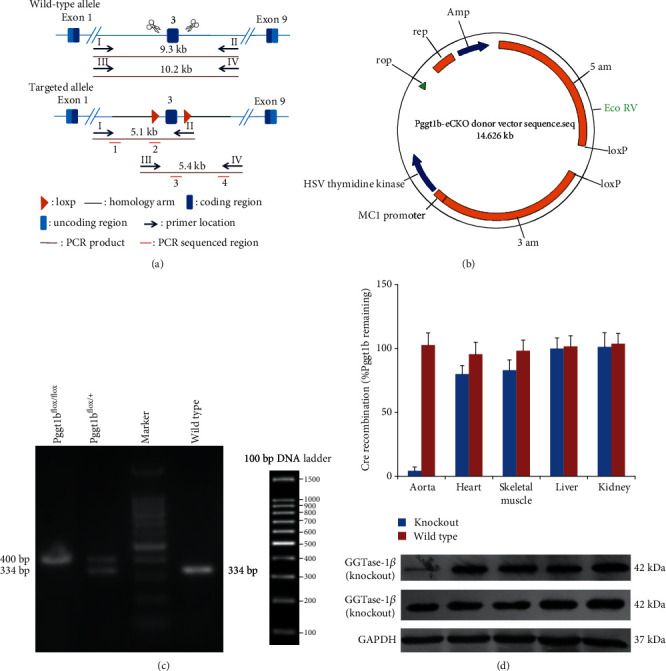
Generation and confirmation of the conditional knockout mice for the *β* subunit of GGTase-1. (a) The strategy for creating Pggt1b^flox^ mice. In homologous recombinant vectors (donor vectors), exon 3 of Pggt1b is flanked by loxP sites (arrowheads). The expression of Cre recombinase results in the excision of exon 3, creating a frameshift mutation and a null allele. The locations of primers for genotyping are indicated. (b) The plasmid map of homologous recombinant vectors (donor vectors). (c) Analysis of different genotypes by different sizes of PCR product fragments. Pggt1b^flox/flox^: one band with 400 bp; Pggt1b^flox/+^: two bands with 334 bp and 400 bp; wild type: one band with 334 bp. (d) GGTase-1 conditional knockout was confirmed by real-time PCR and western blot in different tissues from Pggt1b^*Δ*/*Δ*^ mice.

To produce mice lacking GGTase-I in VSMCs, Pggt1b conditional knockout mice were generated using the CRISPR/Cas9 system. The strategy for creating Pggt1b^flox^ mice is illustrated in Figure 1(a). In the targeted allele (Pggt1b^flox^), loxP sites flank exon 3, which is critical for enzymatic activity. Homologous recombinant vectors (donor vectors) were constructed by In-Fusion cloning and are shown in Figure 1(b). Pggt1b^flox/+^ and Pggt1b^flox/flox^ mice were healthy and fertile. After loxP locus insertion, different genotypes (Pggt1b^flox/+^, Pggt1b^flox/flox^, and wild type) could be distinguished by different sizes of PCR product fragments (Figure 1(c)). To test the conditional knockout of GGTase-I, we detected the RNA and protein expressions in different tissues using real-time PCR and western blotting. As expected, homozygous Pggt1b^*Δ*/*Δ*^ mice eliminated GGTase-I expression in aortic lysates, but not in the heart, skeletal muscle, liver, or kidney (Figure 1(d)). In heterozygous knockout mice (Pggt1b^*Δ*/+^), GGTase-I expression in the aorta was reduced by approximately 40% (data not shown).

### 3.2. Glucose and Lipid Content

As shown in Table 1, both in Pggt1b^*Δ*/*Δ*^ and wild-type mice, STZ and atherogenic diet caused extremely higher FBG than in strain-matched nondiabetic groups. FBG levels were similar between the diabetic wild-type and the diabetic Pggt1b^*Δ*/*Δ*^ mice. Meanwhile, the contents of TC, LDL-C, and TG were also considerably greater in diabetic Pggt1b^*Δ*/*Δ*^ and wild-type mice than in nondiabetic mice. There was no difference in TC, LDL-C, and TG levels between the diabetic wild-type and the diabetic Pggt1b^*Δ*/*Δ*^ mice. However, STZ and atherogenic diet did not affect the levels of HDL-C either in Pggt1b^*Δ*/*Δ*^ or in wild-type mice.

### 3.3. Knockout of VSMC GGTase-I Attenuates Diabetes-Accelerated Atherosclerosis

**Figure 2:**
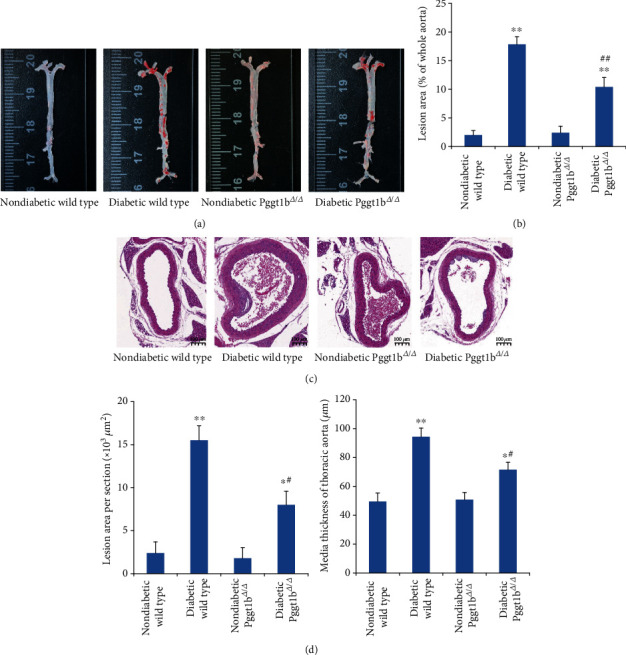
Conditional knockout of VSMC GGTase-I attenuates diabetes-accelerated atherosclerosis. (a) Representative images of en face oil red O staining of aortas. (b) Quantification of oil red O positive areas in en face aortas. (c) Representative hematoxylin and eosin staining (HE) of cross-sectional aortas. (d) Quantification of lesion areas and media thickness from HE staining cross-sectional aortas. Data are expressed as mean ± SEM, *n* = 5 for each group. ^∗^*P* < 0.05 and ^∗∗^*P* < 0.01 versus strain-matched nondiabetic mice. ^#^*P* < 0.05 and ^##^*P* < 0.01 versus diabetic wild-type mice.

The morphometric data of atherosclerotic lesion are summarized in Figure 2. Analysis of en face aortas (Figures 2(a) and 2(b)) indicated that diabetic wild-type mice developed greater atherosclerotic lesions than nondiabetic wild type. Similarly, diabetic Pggt1b^*Δ*/*Δ*^ mice have more lesion burden at the aorta than nondiabetic Pggt1b^*Δ*/*Δ*^. However, the atherosclerotic lesions were remarkably attenuated in diabetic Pggt1b^*Δ*/*Δ*^ mice than in diabetic wild type. Correlated with these findings, analysis of cross-sectional aortas (Figures 2(c) and 2(d)) also revealed that the lesion area and media thickness were notably less in diabetic Pggt1b^*Δ*/*Δ*^ mice than in diabetic wild type.

### 3.4. Knockout of VSMC GGTase-I Reduces the Abundant Hypertrophy of VSMCs in the Atherosclerotic Lesions of Diabetic Mice

**Figure 3:**
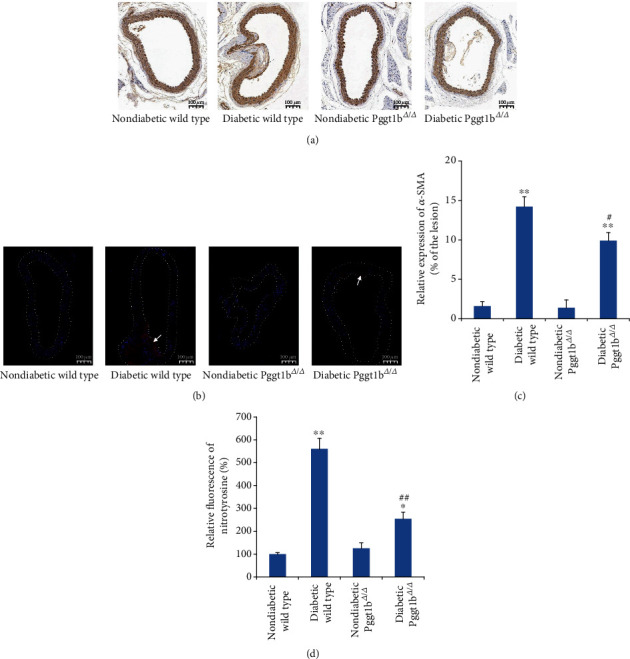
Conditional knockout of VSMC GGTase-I reduces the abundant hypertrophy and excessive oxidative stress of VSMCs in the atherosclerotic lesions of diabetic mice. (a) Representative immunohistochemical analysis of atherosclerotic lesions via staining with *α*-SMA. (b) The immunofluorescence expression of nitrotyrosine (FITC staining, red) was examined in the aortic walls. DAPI counterstaining (blue) indicates nuclear localization. The arrows show the areas with obvious FITC staining. (c) Quantification of the *α*-SMA-positive area in aortic lesions. (d) Relative fluorescence intensity of nitrotyrosine in aortic walls. Data are expressed as mean ± SEM, *n* = 5 for each group. ^∗^*P* < 0.05 and ^∗∗^*P* < 0.01 versus strain-matched nondiabetic mice. ^#^*P* < 0.05 and ^##^*P* < 0.01 versus diabetic wild-type mice.

Next, to ascertain the hypertrophy of VSMCs in the atherosclerotic lesions, *α*-SMA was detected by immunohistochemical staining. As shown in Figure 3, in both diabetic wild-type and diabetic Pggt1b^*Δ*/*Δ*^ mice, the *α*-SMA-positive area in the atherogenic lesions was remarkably increased than in strain-matched nondiabetic groups. However, the *α*-SMA-positive area in the lesions of diabetic Pggt1b^*Δ*/*Δ*^ mice was diminished to 69.6% of that observed in diabetic wild-type mice (Figure 3(c)). In contrast, the extent of *α*-SMA immunoreactivity was similar in the nonplaque area of four groups (data not shown).

### 3.5. Knockout of VSMC GGTase-I Reduces the Excessive Oxidative Stress in the Aortic Wall from Diabetic Mice

Protein nitration, a marker for oxidative stress in the aortic wall, was assessed by immunofluorescence staining with an antibody against nitrotyrosine. As shown in Figure 3, the levels of nitrotyrosine were increased in aortic walls from two diabetic groups when compared with vessels from strain-matched nondiabetic groups. Lack of VSMC GGTase-I caused substantially lower nitrotyrosine levels in diabetic Pggt1b^*Δ*/*Δ*^ aortas than in diabetic wild-type mice (Figures 3(b) and 3(d)).

### 3.6. Knockout of VSMC GGTase-I Inhibits the Proliferation of VSMCs Induced by High Glucose In Vitro

**Figure 4:**
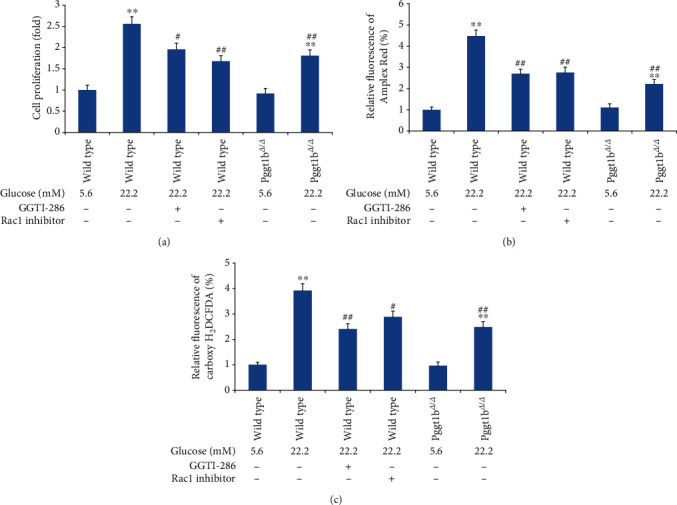
Effects of GGTase-I knockout on high glucose (22.2 mM) induced proliferation and ROS generation of VSMCs. VSMCs were isolated from wild-type and Pggt1b^*Δ*/*Δ*^ mice and treated with normal glucose (5.6 mM) or high glucose (22.2 mM) for 72 h. VSMCs from wild-type mice were also cultured with a selective GGTase-I inhibitor (GGTI-286, 10 *μ*M) or Rac1 inhibitor (100 *μ*M) for 24 h. Cell proliferation was assessed by MTT incorporation assay (a). ROS production in VSMCs was measured by Amplex Red (b) and carboxy-H2DCFDA (c) chemiluminescence, respectively. Data are expressed as mean ± SEM, *n* = 5 for each group. ^∗∗^*P* < 0.01 versus the strain-matched normal glucose group. ^#^*P* < 0.05 and ^##^*P* < 0.01 versus the wild-type high-glucose group.

As expected, VSMCs from both Pggt1b^*Δ*/*Δ*^ and wild-type aortas incubated with high glucose (22.2 mM) resulted in a remarkable increase of cell proliferation than normal glucose (5.6 mM) (Figure 4(a)). However, conditional knockout of VSMC GGTase-I caused a notable reduction of cell proliferation evoked by high glucose than in the wild-type high-glucose group. Similarly, in wild-type VSMCs, coincubation with a selective GGTase-I inhibitor (GGTI-286, 10 *μ*M) or Rac1 inhibitor (100 *μ*M) both reduced the high-glucose-induced VSMC proliferation (*P* < 0.05 and *P* < 0.01 versus the wild-type high-glucose group, respectively).

### 3.7. Knockout of VSMC GGTase-I Inhibits the Excessive ROS in VSMCs Induced by High Glucose In Vitro

ROS production (H_2_O_2_) in VSMCs was measured by Amplex Red and carboxy-H_2_DCFDA chemiluminescence, respectively, and the results obtained by these two different approaches were almost consistent (Figures 4(b) and 4(c)). Both Pggt1b^*Δ*/*Δ*^ and wild-type VSMCs cultured under high glucose (22.2 mM) showed greater ROS levels when compared to normal glucose (5.6 mM) treated strain-matched VSMCs, whereas the ROS level in Pggt1b^*Δ*/*Δ*^ VSMCs treated with high glucose was dramatically decreased than in wild-type VSMCs treated with the same high glucose. Additionally, in wild-type VSMCs, coincubation with a selective GGTase-I inhibitor (GGTI-286, 10 *μ*M) or Rac1 inhibitor (100 *μ*M) both attenuated the high-glucose-evoked excessive ROS levels.

### 3.8. Knockout of VSMC GGTase-I Inhibits the Activation of Small GTPase, Rac1

**Figure 5:**
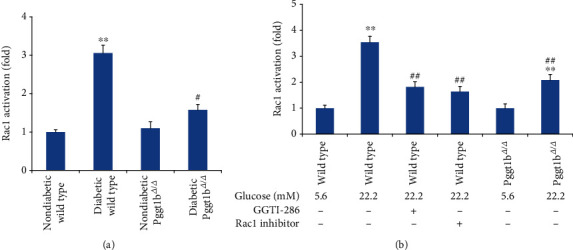
Conditional knockout of VSMC GGTase-I inhibits the activation of small GTPase, Rac1, both in vivo and in vitro. (a) Rac1 activities were determined in aortas from four groups. (b) VSMCs were isolated from wild-type and Pggt1b^*Δ*/*Δ*^ mice and treated with normal glucose (5.6 mM) or high glucose (22.2 mM) for 72 h. VSMCs from wild-type mice were also cultured with selective GGTase-I inhibitor (GGTI-286, 10 *μ*M) or Rac1 inhibitor (100 *μ*M) for 24 h. Rac1 activities were measured by the Rac1 G-LISA Activation Assay Kit according to the manufacturer's instruction. Data are expressed as mean ± SEM, *n* = 5 for each group. ^∗∗^*P* < 0.01 versus strain-matched nondiabetic mice (or strain-matched normal glucose VSMCs). ^#^*P* < 0.05 and ^##^*P* < 0.01 versus diabetic wild-type mice (or wild-type high-glucose VSMCs).

The activation of small GTPase, Rac1, depends on its conversion from the GDP- to GTP-bound state and the membrane location via the process of protein geranylgeranylation by GGTase-I [6]. The levels of GTP-bound active form of Rac1 in aortas and cultured VSMCs were determined by G-LISA kits. In vivo, diabetic wild-type mice exhibited a remarkably higher Rac1 activity than nondiabetic wild-type, whereas a 16-week duration of diabetes had no obvious effect on aortic Rac1 activity in Pggt1b^*Δ*/*Δ*^ mice (Figure 5(a)). In other words, the Rac1 activity in diabetic Pggt1b^*Δ*/*Δ*^ mice was much lower than in the diabetic wild-type aorta. In vitro, wild-type VSMCs treated with high glucose (22.2 mM) showed a significantly greater level of Rac1 activity (*P* < 0.01 vs. wild-type normal glucose VSMCs), while coincubation with a selective GGTase-I inhibitor (GGTI-286, 10 *μ*M) or Rac1 inhibitor (100 *μ*M) restrained this increase of Rac1 activity evoked by high glucose (Figure 5(b)). Similarly, Rac1 activity in Pggt1b^*Δ*/*Δ*^ VSMCs treated with high glucose was much lower than wild-type VSMCs treated with the same high glucose (Figure 5(b)). However, either in vivo or in vitro, high glucose had no effect on the expression of total Rac1 in all groups mentioned above (data not shown).

### 3.9. Knockout of VSMC GGTase-I Suppresses the Membrane Localization of p47phox and NOXO1

**Figure 6:**
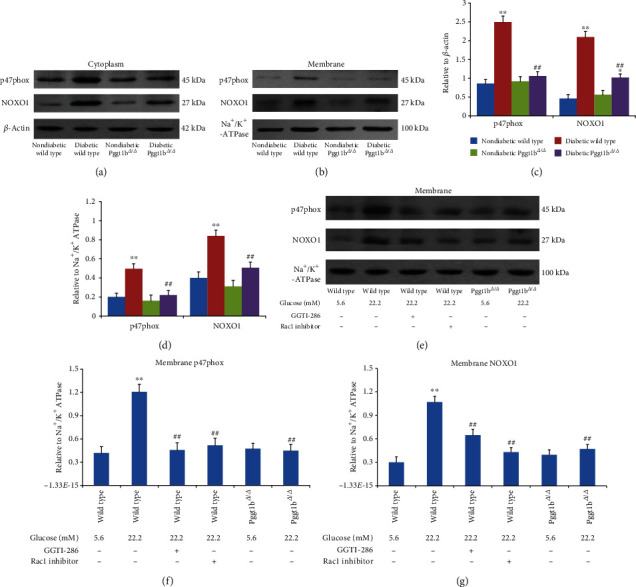
Effects of GGTase-I knockout on p47phox and NOXO1 in cytosolic and membrane fractions from cultured VSMCs in vitro and aortic tissue in vivo. The protein expressions were detected by western blot analysis. (a) Representative blots and (c) densitometric average in membrane fractions from aortic tissue in vivo. (b) Representative blots and (d) densitometric average in cytosolic fractions from aortic tissue in vivo. (e) Representative blots and (f, g) densitometric average in membrane fractions from cultured VSMCs in vitro. VSMCs were isolated from wild-type and Pggt1b^*Δ*/*Δ*^ mice and treated with normal glucose (5.6 mM) or high glucose (22.2 mM) for 72 h. VSMCs from wild-type mice were also cultured with a selective GGTase-I inhibitor (GGTI-286, 10 *μ*M) or Rac1 inhibitor (100 *μ*M) for 24 h. *β*-Actin and Na^+^/K^+^-ATPase were used as loading controls for the cytoplasm and plasma membrane, respectively. Data are expressed as mean ± SEM, *n* = 5 for each group. ^∗^*P* < 0.01 and ^∗∗^*P* < 0.01 versus strain-matched nondiabetic mice (or strain-matched normal glucose VSMCs). ^##^*P* < 0.01 versus diabetic wild-type mice (or wild-type high-glucose VSMCs).

NOX is the main source of ROS in the vasculature [16–19]. Of the various NOX isoforms, NOX1 and NOX2 are the most important ones in VSMC proliferation [16–19]. These enzymes consist of a membrane-bound NOX isoforms (NOX1 and NOX2) and several cytosolic regulatory subunits. p47phox and NOXO1 are cytoplasmic subunit for NOX1 and NOX2, respectively [16–19]. The activation of NOX is initiated by translocation of the cytoplasmic components, involving p47phox, NOXO1, and Rac1, to the membrane [16–19]. In our study, hyperglycemia had no effect on the expressions of membrane-bound NOX isoforms (NOX1 and NOX2) in vivo or in vitro in all groups (data not shown). For cytosolic subunits (p47phox and NOXO1), the situation is quite different. In vivo data showed that the expression of p47phox and NOXO1 was significantly increased in both membrane and cytoplasmic fractions of aortas from diabetic wild-type mice, when compared to nondiabetic wild type (Figures 6(a)–6(d)). These upregulations of p47phox and NOXO1 also happened in aortas from diabetic Pggt1b^*Δ*/*Δ*^ mice (*P* < 0.01 versus nondiabetic Pggt1b^*Δ*/*Δ*^ mice), whereas these increases were greatly inhibited when compared to diabetic wild-type mice (Figures 6(a)–6(d)). In vitro, wild-type VSMCs treated with high glucose (22.2 mM) exhibited a much higher level of membrane p47phox and NOXO1 expression (both *P* < 0.01 vs. wild-type normal glucose VSMCs), while coincubation with a selective GGTase-I inhibitor (GGTI-286, 10 *μ*M) or Rac1 inhibitor (100 *μ*M) restrained this increase (Figures 6(e)–6(g)). Meanwhile, membrane p47phox and NOXO1 expression in Pggt1b^*Δ*/*Δ*^ VSMCs under high glucose was dramatically lower than wild-type VSMCs treated with the same high glucose (Figures 6(e)–6(g)). However, in either wild-type or Pggt1b^*Δ*/*Δ*^ VSMCs, cytoplasmic p47phox and NOXO1 expressions did not change in response to high glucose, GGTI-286, or Rac1 inhibitor (data not shown).

### 3.10. Knockout of VSMC GGTase-I Attenuates the Phosphorylation of ERK1/2 and JNK

**Figure 7:**
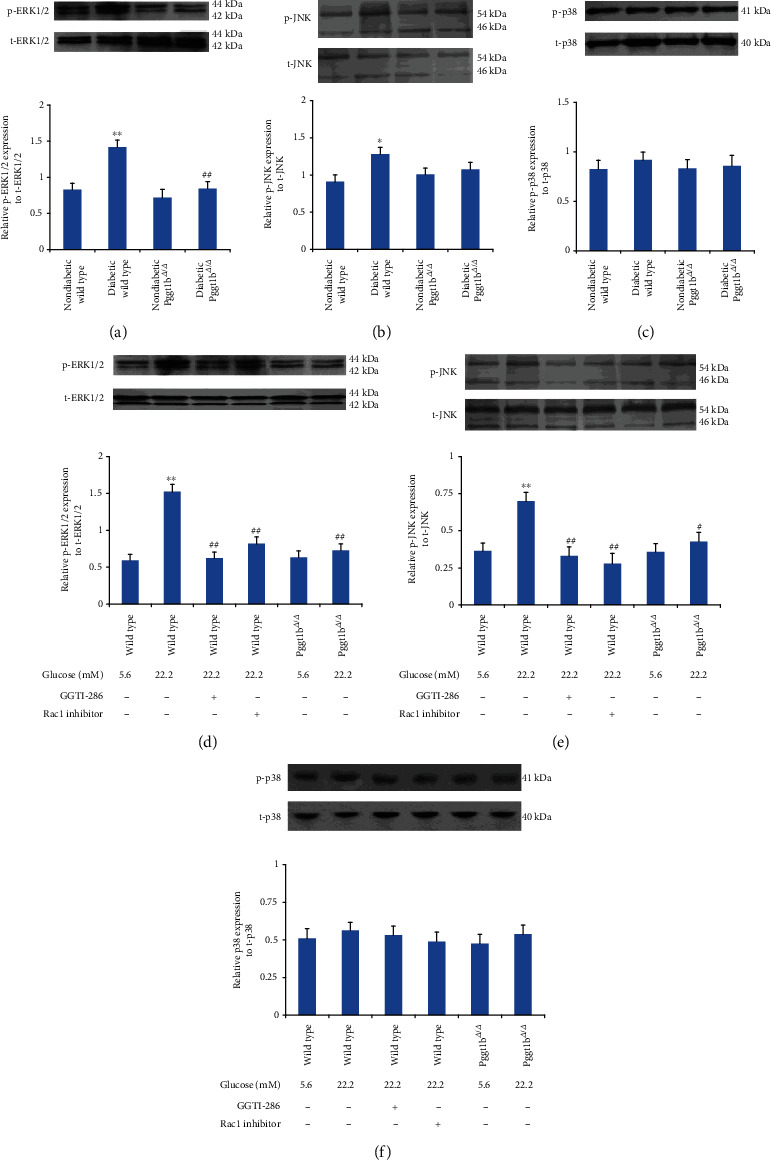
Effects of GGTase-I knockout on the phosphorylation of ERK1/2, JNK, and p38 in cultured VSMCs in vitro and aortic tissue in vivo. Western blot analysis of ERK1/2 (a), JNK (b), and p38 (c) phosphorylation and total protein expression in aortic tissue from four groups. Western blot of ERK1/2 (d), JNK (e), and p38 (f) phosphorylation and total protein expression in cultured VSMCs in vitro. VSMCs were isolated from wild-type and Pggt1b^*Δ*/*Δ*^ mice and treated with normal glucose (5.6 mM) or high glucose (22.2 mM) for 72 h. VSMCs from wild-type mice were also cultured with a selective GGTase-I inhibitor (GGTI-286, 10 *μ*M) or Rac1 inhibitor (100 *μ*M) for 24 h. *β*-Actin was used as an internal control. Data expressed as mean ± SEM, *n* = 5 for each group. ^∗^*P* < 0.01 and ^∗∗^*P* < 0.01 versus strain-matched nondiabetic mice (or strain-matched normal glucose VSMCs). ^#^*P* < 0.05 and ^##^*P* < 0.01 versus diabetic wild-type mice (or wild-type high-glucose VSMCs).

To investigate the effects of GGTase-I knockout on the MAPK signaling pathway, the phosphorylated protein levels of extracellular signal-regulated kinase (ERK1/2), c-Jun N-terminal kinase (JNK), and p38 MAPK were measured by western blot (Figure 7). The protein expressions of phospho-ERK1/2 and phospho-JNK were markedly increased in aortas from diabetic wild-type mice than nondiabetic wild type. However, these increases were eliminated in diabetic Pggt1b^*Δ*/*Δ*^ mice when compared to diabetic wild-type mice (Figures 7(a) and 7(b)). Likewise, in wild-type VSMCs cultured in vitro, the expressions of phospho-ERK1/2 and phospho-JNK were notably increased by incubation with 22.2 mM glucose, and these effects were attenuated by pretreatment with a selective GGTase-I inhibitor (GGTI-286, 10 *μ*M) or Rac1 inhibitor (100 *μ*M) (Figures 7(d) and 7(e)). Then, in Pggt1b^*Δ*/*Δ*^ VSMCs cultured with 22.2 mM glucose, phospho-ERK1/2 and phospho-JNK expressions were dramatically lower than in wild-type VSMCs treated with the same high glucose (Figures 7(d) and 7(e)). However, the expressions of total ERK1/2 and total JNK did not change among all groups mentioned above (Figures 7(a), 7(b), 7(d), and 7(e)). Besides, there was no difference of phospho-p38 and total p38 expressions among all groups mentioned above (Figures 7(c) and 7(f)), especially between diabetic wild-type and diabetic Pggt1b^*Δ*/*Δ*^ mice in vivo, between wild-type and Pggt1b^*Δ*/*Δ*^ VSMCs treated with high glucose in vitro.

## 4. Discussion

Our experiments provide strong evidence of a major pathophysiological role for GGTase-I in diabetes-accelerated atherosclerosis. We found that GGTase-I deficiency in VSMCs markedly reduced the lesion burden in an experimental mouse model of atherosclerosis secondary to hyperglycemia and hyperlipemia. Furthermore, in vivo and in vitro studies demonstrated that this protective effect must be related to inhibition of VSMC proliferation, and potential mechanisms probably involved interfering Rac1, p47phox, and NOXO1 membrane localization, inhibiting ROS generation, and decreasing ERK1/2 and JNK phosphorylation.

Diabetes-accelerated atherosclerosis is a complex pathological process, which plays a pivotal role in the progression of cardio-, cerebro-, and peripheral vascular diseases, accounting for the major mortalities and disabilities of diabetic patients [5, 20–22]. How diabetes promotes the pathogenesis of atherosclerosis is not completely understood and probably has a multifactorial origin. It has been widely accepted that accelerated proliferation of VSMCs is the fundamental event in the development of atherosclerosis in diabetes [4, 23]. In our previous study, high glucose (22.2 mM) remarkably induced the VSMC proliferation and GGTase-I upregulation in vitro and in vivo [12]. Then, in our present experiments, Pggt1b^*Δ*/*Δ*^ mice were induced diabetes by STZ and an atherogenic diet. As expected, diabetic Pggt1b^*Δ*/*Δ*^ mice exhibited accelerated atherosclerotic lesions, but the lesion burden was remarkably reduced versus diabetic wild-type mice regardless of the similar levels of glucose and lipid. Additionally, genetic knockout of GGTase-I inhibited the proliferation of VSMCs in both atherosclerotic lesions and high-glucose cultured cells. All these findings excitingly suggested the important role of GGTase-I in VSMC proliferation during diabetes-accelerated atherosclerosis, but mechanisms involved were not clear.

Oxidative stress mediated by ROS plays a key role in the pathogenesis of diabetes-accelerated atherosclerosis. The probable mechanisms include endothelial cell dysfunction, monocyte/macrophage recruitment and activation, stimulation of inflammation, and VSMC migration and proliferation [24–26]. In our present study, GGTase-I deficiency in VSMCs caused a significant decrease of excessive oxidative stress in diabetic aortas. In vitro, excessive ROS production evoked by high glucose was also notably inhibited in cultured Pggt1b^*Δ*/*Δ*^ VSMCs. Both results confirmed that genetic knockout of GGTase-I protected VSMCs from oxidative injury under diabetic state, and further exploration was needed.

NOX has been implicated as the main source of vascular ROS generation in response to high glucose [16–19]. Seven distinct isoforms of this enzyme have been identified, of which NOX1 and NOX2 are the most important ones in VSMC proliferation [16–19]. These enzymes consist of a membrane-bound heterodimer (NOX1 and NOX2) and several cytosolic regulatory subunits, involving NOXO1 for NOX1, p47phox for NOX2, and small GTP-binding protein Rac1 [27, 28]. The activation of NOX is initiated by translocation of the cytoplasmic components to the membrane [16–19]. As one of the important factors initiating NOX assembly, Rac1, its membrane localization and activation depends on the process of protein geranylgeranylation by GGTase-I [29–31]. In our experiments in vitro and in vivo, GGTase-I deficiency in VSMCs inhibited Rac1 activation stimulated by hyperglycemia but had no effect on the expression of total Rac1, proving in turn that knockout of GGTase-I only affected the activating process of small GTP-binding protein Rac1, possibly via inhibition of its geranylgeranylation. Moreover, we also found that lack of VSMC GGTase-I in vivo suppressed the upregulation of membrane p47phox and NOXO1 induced by hyperglycemia, and the situation was almost the same in the cytoplasm. But high glucose had no effect on the expressions of NOX1 and NOX2 in vivo or in vitro in all groups. Taken together, these results further supported the hypothesis that GGTase-I deficiency prevented Rac1 geranylgeranylation and inhibited the assembly of NOX1 and NOX2 cytosolic regulatory subunits, which subsequently led to the decrease of vascular ROS generation.

The signaling molecular by which GGTase-I deficiency exerts an antiatherosclerotic effect in diabetes remains unclear. MAPKs are serine-threonine kinases that mediate intracellular signaling responding to varieties of stimuli including ROS [32–34]. As is well known, the MAPK family is composed of ERK1/2, JNK, and p38. A growing body of evidence has demonstrated that ROS-ERK1/2 cascades participate in the regulation of VSMC proliferation in vitro and neointima formation in vivo [35–37]. Previous studies also showed that JNK, p38, and ERK1/2 phosphorylation acted a critical role in high-glucose-induced oxidative injury and VSMC proliferation [38, 39]. In our in vivo study, we found that lack of VSMC GGTase-I reduced the hyperphosphorylation of ERK1/2 and JNK stimulated by diabetes. In line with in vivo results, ERK1/2 and JNK phosphorylation was inhibited in high-glucose-treated VSMCs with GGTase-I knockdown. However, different from the previous experiments, the phosphorylation of p38 in our study was of no significant change. Therefore, based on these results, our presumption was that GGTase-I deficiency protected proliferative VSMCs from oxidative injury under diabetic state, probably via ERK1/2 and JNK pathways, but not p38.

Furthermore, in our study, VSMCs from wild-type mice were also cultured in the presence of a selective GGTase-I inhibitor or Rac1 inhibitor. Then, it was observed that both inhibitors notably restrained high-glucose-evoked upregulation of VSMC proliferation, ROS content, Rac1 activity, membrane p47phox and NOXO1 expression, and phospho-ERK1/2 and phospho-JNK level. These results are very consistent with the above data from Pggt1b^*Δ*/*Δ*^ VSMCs stimulated by high glucose, indicating that the geranylgeranylation of Rac1 mediated by GGTase-I is probably responsible for the antiatherosclerotic effect in our study.

## 5. Conclusions

**Figure 8:**
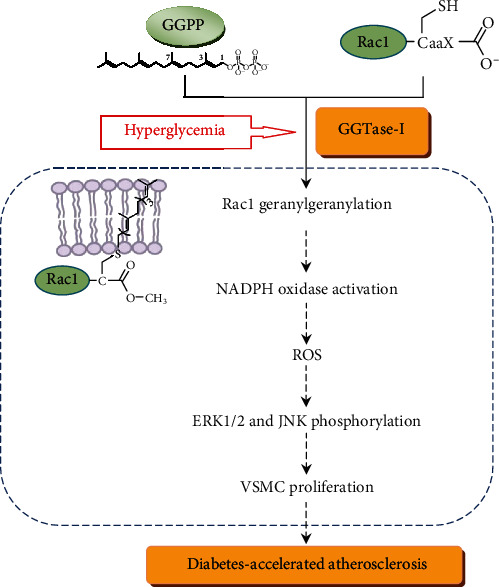
The role of GGTase-I in diabetes-accelerated atherosclerosis and the probable mechanisms are summarized. GGPP: geranylgeranyl pyrophosphate.

Our work provided the experimental evidences that GGTase-I knockdown efficiently blocked diabetes-accelerated atherosclerosis, and this protective effect must be related to inhibition of VSMC proliferation. The potential mechanisms probably involved interfering Rac1 geranylgeranylation, inhibiting p47phox and NOXO1 membrane localization, reducing ROS generation, and decreasing ERK1/2 and JNK phosphorylation (summarized in Figure 8). Although there are many essential differences between experimental and clinical studies, our study represents a potentially promising therapeutic strategy for the treatment of diabetic macrovascular disease in the future.

## Figures and Tables

**Table 1:** Glucose and lipid analysis after a 16-week diabetic duration in Pggt1b^*Δ*/*Δ*^ or wide-type mice.

Group	FBG (mM)	TC (mM)	HDL-C (mM)	LDL-C (mM)	TG (mM)
Wild type					
Nondiabetic	6.44 ± 0.75	1.88 ± 0.08	0.90 ± 0.06	0.47 ± 0.07	1.42 ± 0.07
Diabetic	21.86 ± 1.10^∗∗^	2.56 ± 1.10^∗∗^	0.84 ± 0.07	0.84 ± 0.07^∗^	2.14 ± 0.07^∗∗^
*Pggt1b^Δ/Δ^*					
Nondiabetic	6.30 ± 0.61	1.86 ± 0.07	0.90 ± 0.09	0.43 ± 0.17	1.33 ± 0.06
Diabetic	23.34 ± 1.41^∗∗^	2.55 ± 0.08^∗∗^	0.93 ± 0.06	0.91 ± 0.09^∗∗^	2.09 ± 0.09^∗∗^

Data are expressed as mean ± SEM, *n* = 5 for each group. ^∗^*P* < 0.05 and ^∗∗^*P* < 0.01 versus strain-matched nondiabetic mice. FBG: fasting blood glucose; TC: total cholesterol; HDL-C: high-density lipoprotein cholesterol; LDL-C: low-density lipoprotein cholesterol; TG: triglyceride.

## Data Availability

The data used to support the findings of this study are available from the corresponding author upon request.
